# Supplementary material for: A Critical View of the Application of the APEX Software (Aqueous Photochemistry of Environmentally-Occurring Xenobiotics) to Predict Photoreaction Kinetics in Surface Freshwaters
Source: Molecules. 2019 Dec 18;25(1):9. doi: 10.3390/molecules25010009 (PMC7017383; doi:10.3390/molecules25010009)
Supplement: Supplementary file 1 [file molecules-25-00009-s001.zip › 3.2.4_gcc-4.4.0.zip!/3.2.4_gcc-4.4.0/3.2.4_gcc-4.4.0/doc/octave/html/interpreter/Area-series.html]

Area series - Untitled


Next: Bar series,
Previous: Data sources in object groups,
Up: Object Groups


---

##### 15.2.8.2 Area series

Area series objects are created by the `area` function. Each of the
`hggroup` elements contains a single patch object. The properties
of the area series are

`basevalue`: The value where the base of the area plot is drawn. `linewidth` `linestyle`: The line width and style of the edge of the patch objects making up the areas. See Line Styles. `edgecolor` `facecolor`: The line and fill color of the patch objects making up the areas. See Colors. `xdata` `ydata`: The x and y coordinates of the original columns of the data passed to `area` prior to the cumulative summation used in the `area` function. `xdatasource` `ydatasource`: Data source variables.
